# Sorted: an experimental interpretive poetry piece on injectable medications care at the end of life

**DOI:** 10.1177/17449871251357097

**Published:** 2025-09-02

**Authors:** Bella Madden, Ben Bowers

**Affiliations:** Public Contributor, Department of Public Health and Primary Care, University of Cambridge, Cambridge, UK; Assistant Professor of Primary Care and Honorary Nurse Consultant in Palliative Care. Department of Public Health and Primary Care, University of Cambridge, Cambridge, UK; Community Nursing Research Forum Lead, Queen’s Institute of Community Nursing, UK

**Sorted.**

It’s you and me love, so please

keep me in the loop . . . promise?

You see, I’ve got it sorted now:

have the number – just in case -

(and that nurse is great eh?

I’m sure she’ll keep us safe -

point us the right way -

and she’s only a phone call away).

Wait – I’ll get that love – sit back down.

There. Good. Comfy?

Where was I? Oh yes – that nurse -

she knows exactly what to say, doesn’t she?

And so little delay – unlike the GP

and the hospital and God forbid A & E.

That was a long haul huh ? Telling the story

over and over and having to fight. . .

. . .no. Sit tight. I’ll pass it.

How’s the pain love? OK. But don’t go

all stoic on me. Promise?

Right. So. Let’s keep her on side.

It was sort of scary that last time

chasing your meds all over the place

thank gooodness that chemist was open

and I’d left you alone for two hours by then!

I know. I know you were ok. But I worry

when I have to disappear.

Don’t leave here.

Anyway – those meds are safely tucked in the cupboard now.

We’re sorted. All under control.

Oh yes! I’ll just check the date on them.

Thanks for the reminder.

Oh, OK. Not to worry -

I’ll go fetch the wipes and a fresh bag,

I left them upstairs last night. Sorry.

. . . . . . . . . . . . . . . . . . .there. . . . . . .that’s it I think

. . . . . . . . .easy. . . . . . . .let me just. . . . . . . . .right. . .

now ease back slowly. Damn. Sorry. . . .

I forgot to tape that bit up. Here we go. . . . . .

There. You OK? Sure? Good. . . . .done.

I’ll make a cuppa. Want the telly back on?

Hey love. We’ve got this bit sorted eh?

Ha ha!! Last week – yes, was it? Gosh. Was

it only last week? Crikey. Last week we had no idea!

Now. Well. Just look at us. Quite a team.

Yes. No problem. I’ll turn it off,

you have a sleep. Wait – I’ll get those cushions

sorted. I’ve got bits to do too - need to

get some more dressings and pads.

I’ll be in the back room. OK?

Don’t go anywhere.

. . . . . . . . . . . . . . . . . . . . . . . . . . . . . . . . . . .love??

Hello sweetheart. No. Nothing wrong but

. . .well. . . . . . . .you were moaning. . . .

should I call the nurse? Yes. I think so too.

It does seem to be getting worse.

Done. Hopefully not too long.

Here. Hold my hand. Hang on. . . . . . . . .

                    Oh, hi there. Hello. Oh Jeanie, I’m so glad it’s you. Come in. Tea?

                         Yes. No, we’ve done that but it’s just not working as it

                           did last week – and I’m so sorry to bother you

                            but we – well, I, – was worried and actually

                              Jeanie – scared. Thank you. Thank you.

                                Gosh – that’s made me feel better.

                                 I thought I’d done it wrong.

                                   I’m OK. Just tears.

                                    Relief really.

                                     It’s ok.

You awake love?

Sorry love, sorry –no, I can’t hear?

Oh my love, oh my dear -

Yes. We need those meds.

I’ll fetch them now and phone the nurses.

It’s ok. Jeanie’s here.

## Being asked to write the poem

‘Sorted’ is a response to reading research interview transcripts with family caregivers, patients and clinical staff involved in using injectable end-of-life symptom management medicines. These are a pack of medicines that are routinely prescribed for dying people in the United Kingdom and left in the home ‘just in case’ of emergencies (Bowers et al., 2024). The pack often contains powerful medicines, including morphine for pain and midazolam as a sedative for distress (Bowers et al., 2022). In my capacity as a lay member of the Palliative and End of Life Care Group in Cambridge (PELiCam), I had been offered the chance to read some of this ‘raw’ data in order to contribute my own ‘first level’ analysis of the main issues arising *for me*. This I had done and I had enjoyed this chance to identify the threads of similarity as I perceived them between the various conversations.

Therefore, when Ben (researcher) first mooted the idea of a poetic response from me, I was cautiously enthusiastic. The enthusiasm arose from the opportunity to write creatively to a loose ‘brief’ about an area of life I had experience of. The caution was rooted in confusion – why would this be wanted in the seriously academic world of qualitative healthcare research? I felt uneasy because I could not really ‘get my head around’ what it (and I in this case) was ‘for’. It really was not until a meeting with Ben and Sarah (journal editor) that I got it: this was a genuine opportunity to offer an honest response from outside academia to a part of the research – namely, raw data. This was not a self-audit tick box either but genuine (that word again) collaboration between a researcher and ‘an interested member of the public’. Furthermore, that response itself was not to be swallowed whole into the data analysis, but allowed simply to ‘be’ – in its own space. If you are a researcher reading this, could this be of interest to you?

The time lag between the initial reading and the subsequent writing was a few months, and I really struggled to find a way in. My initial reactions to the transcripts had been relayed to the research team by email in a fairly traditional PowerPoint format – basically my interpretation of issues and dynamics either as I recognised them from my own experience (as a family caregiver in the past) or as they repeated across interviews and revealed themselves that way. I was responding to data with a basic first-level analysis and from a lay perspective, as asked.

When it came therefore to drafting the poem, I turned back to that PowerPoint and highlighted what I thought to be the strongest issues, intending to use those as a way in and the basis of some sort of structure. I did not want to re-read the interviews themselves, as I was conscious of the need to protect the privacy of the stories they contained and so abstracting those stories to ‘issues’ seemed the way to go. But I still found myself unable to get started. Somehow, it all felt too contrived.

So eventually, pressed by an agreed deadline, I just opened the laptop, stopped thinking and started writing with the first moment or phrase that came to mind. What you see here is very nearly exactly as it was first written bar a few minor alterations.

As I wrote, I visualised a heterosexual married couple of middle age: she is terminally ill and he is her carer. I was not visualising my own experience, but the end result *felt* like it. There is a saying I use often – ‘I write to find out what I am thinking’. It has been striking to me on many occasions that whatever I am writing (particularly poetry) will reveal to me on its completion all sorts of metaphors and connections that I was not consciously aware of, but which, once revealed, help me to better understand an event or situation. What was interesting to me about *this* process was the *feel* of what emerged. Poetically, it is ropey, but the feel of it: nervous, edgy, the subject constantly self-checking and in need of reassurance, is deeply familiar. I had not started writing, however, with any conscious desire to express anxiety and edginess. And so, this was ‘writing to find out what I am *feeling*’ (rather than thinking): the suppressed panic as the carer desperately tries to keep the ‘dying’ under control and utter responsibility for a process he does not understand is present in all the repetition, the self-reassurance, the ‘other’ reassurance, the questioning, the assertions, the ‘it’s ok’ and ‘sorted’, and in his palpable relief when Jeanie, the community nurse, arrives.

The end product feels staccato, clumsy, hesitant, repetitive, almost breathless. Discussing this feeling later with Ben, I am suddenly taken back to a morning stuck in a traffic jam. I had nipped out whilst my terminally ill partner slept, only to find myself stuck in a car park queue and unable to drive the couple of miles home as quickly as I wished. Terrified that my partner would die suddenly whilst I was out (although he was not yet end-of-life), I ran into all the shops on the retail park demanding that one of them open up a side entrance. No one seemed to have the key.

You are a caregiver. It is your job to fix. As the physical and social world of the patient shrinks ever smaller, you are the one charged with maintaining a highly functional link with the rest of the vibrant living world: a world that knows nothing of dying. Those few clinicians who support you and who DO know are therefore all the more vital. Particularly if they know you and your story. They can hold your story within ‘the system’ and be a voice for you. You will forgive them almost anything as long as they eventually show up and take your hand. And so, the end-of-life injectable medications – vital though they may be in some circumstances – are only a part of the team required to support a dying person and family caregiver.

So, what underscores or informs all of the ‘themes’ emerging for me, both from my original reading of raw interview data and from the piece above, is the almost total lack of what I want to call ‘community knowledge’ of the actual process of dying. Of what happens when a body dies. Of the ways in which it stops working over time. Of the consequences of that stopping. Of the sequence of signs. Of the utter isolation of that dying from the social world, which, for the caregiver, makes ‘fitting back in’ afterwards problematic. This everyday event – or series of events in each individual case – is hidden from our view and only experienced by the vast majority of us when it is the dying of a family member that is happening. Even then, we may at best witness only brief moments along the way.

I can only speak for the family caregiver, never having been the patient; this loneliness is not only a wider social isolation but also potentially an acutely intimate one. A family caregiver is learning a new role, often under great duress. That new role inevitably edges out the old in terms of the relationship with the dying which can deny or make more remote the comfort of that relationship. If your partner is dying at home, you are now so much more than their partner, and your primary responsibility is to be their carer. The succour that your ‘couple-dom’ once gave you, which cushioned the hard stuff, may well no longer be available and in addition you are witnessing them leaving you.

If I had to animate that process of leaving, it would be a Venn diagram with two circles overlapping to reveal a central shared space. But the circles are slowly, inexorably moving away from each other, and the shared space is shrinking. I *felt* this in the interview transcripts, and it resonated with me as I recalled the increasing acceptance or surrender to dying from my partner just as I desperately tried to keep everything around us safe and still and unchanged.

But you can’t. Nothing stays still and unchanged and neither is the trajectory of change steady. Our carer is astounded by what he has learnt to do and handle in such a short space of time and how quickly new situations become normalised. ‘Crikey. Last week we had no idea!’ he exclaims and is surprised by this revelation because last week was a whole crisis and response away. I felt this from the transcripts, and I recall it from life: periods of intense crisis that required new learning followed by adjusting to and ‘making ok’ whatever new situation had come into play. As time passed, those crises seemed to occur closer and closer together until the exhale of ‘we’re coping’ was always countered by the anxiety of anticipating the next moment when you wouldn’t be.

But Jeanie knows all about that. She’s seen it before, many times, and so the safety you feel when you are in her hands stems from her knowledge, and from your knowing that she knows.

## Ben’s response as a researcher

Reading this poem also made me anxious and edgy, but in the desired way. I could feel the duality of the fictitious family carer providing reassurance to their loved one whilst being full of self-doubt and living in increasingly parallel social worlds. Bella’s response to what was a very open brief is raw, powerful, and full of interpretations that I as a ‘trained’ applied researcher and experienced community nurse could not bring. What you read is authentic, edgy, and thought-provoking. Codifying data from multiple patient-centred cases inevitably loses this interpretative edginess during the process. Both ways work, they have different motives and research outputs.

The poem and process we present here is unashamedly experimental. I wanted to do something different, rather than asking Bella to become an apprentice researcher and learn our way of thinking and doing. The aim was to draw upon Bella’s creative skills and lived experience in interpreting what she read and *felt* from the transcripts. Perhaps we should pay closer attention to what we feel as we interpret data as researchers too and bring this more centrally into our own analyses (Lustick et al., 2024)?

I met with Bella to have a recorded conversation on the process after reading the poem, to help us both shape this written piece. I shared my worries that reading the transcripts may have brought up difficult memories or been hard for Bella to do as someone with lived experience (Fincham et al., 2008). Actually, Bella responded ‘why would you think that?’ Indeed, she was far less affected by the challenging narratives shared than I was as an applied researcher who had interviewed and got to know the people involved over time. She embraced the opportunity to hear people’s shared accounts, keen to learn perspectives, what happened next and how people navigated care at the end of life. Perhaps we need to consider our own naturally paternalistic stances when working with public contributors? I took the decision to let Bella ‘own’ this written piece – I am but the supporting act. Another overt decision, brave maybe, certainly more interesting and impactful for it.

We hope this experimental interpretive piece is worth pondering and thinking over as researchers. We welcome responses.

## References

[bibr1-17449871251357097] Bowers B Antunes B Etkind S , et al Anticipatory prescribing in community end of life care: Systematic review and narrative synthesis of the evidence since 2017. BMJ Supportive & Palliative Care 2024; 8: e612–e623. 10.1136/spcare-2022-004080 PMC10850730 37236648

[bibr2-17449871251357097] Bowers B Pollock K Barclay S. Simultaneously reassuring and unsettling: A longitudinal qualitative study of community anticipatory medication prescribing for older patients. Age and Ageing 2022; 51: afac293. 10.1093/ageing/afac293 PMC9729004 36477784

[bibr3-17449871251357097] Fincham B Scourfield J Langer S. The impact of working with disturbing secondary data: Reading suicide files in a coroner’s Office. Qual Health Res 2008; 18: 853–862. 18503026 10.1177/1049732307308945

[bibr4-17449871251357097] Lustick H Yang X Hakouz A. The role of emotions in qualitative analysis: Researchers’ perspectives. TQR 2024; 29: 1103–1124.

